# Evaluation of 1β-Hydroxylation of Deoxycholic Acid as a Non-Invasive Urinary Biomarker of CYP3A Activity in the Assessment of Inhibition-Based Drug–Drug Interaction in Healthy Volunteers

**DOI:** 10.3390/jpm11060457

**Published:** 2021-05-24

**Authors:** Xue-Qing Li, Roslyn Stella Thelingwani, Leif Bertilsson, Ulf Diczfalusy, Tommy B. Andersson, Collen Masimirembwa

**Affiliations:** 1 Drug Metabolism and Pharmacokinetics, Research and Early Development, Cardiovascular, Renal and Metabolism, BioPharmaceuticals R&D, AstraZeneca, 431 83 Gothenburg, Sweden; Xueqing.Li@astrazeneca.com (X.-Q.L.); tommy.b.andersson@gmail.com (T.B.A.); 2 Department of Pharmaceutical Medicine, African Institute of Biomedical Science and Technology (AiBST), Block C, Wilkins Hospital Complex, Harare, Zimbabwe; rthelingwani@aibst.edu.zw; 3 Division of Clinical Pharmacology-C1:68, Department of Laboratory Medicine at Karolinska Institutet, Karolinska University Hospital, SE-141 86 Stockholm, Sweden; leif.bertilsson@ki.se; 4 Division of Clinical Chemistry, Department of Laboratory Medicine at Karolinska Institutet, Karolinska University Hospital (Huddinge), SE-141 86 Stockholm, Sweden; ulf.diczfalusy@ki.se

**Keywords:** CYP3A, biomarker, human, deoxycholic acid, 1β-hydroxy-deoxycholic acid, urine, midazolam, drug–drug interaction

## Abstract

In this study, we aimed to evaluate the utility of endogenous 1β-hydroxy-deoxycholic acid/total deoxycholic acid ratio (1β-OH-DCA/ToDCA) in spot urine as a surrogate marker of cytochrome P450 3A (CYP3A) activity in the assessment inhibition-based drug–drug interactions in healthy volunteers. This was accomplished through an open-label, three-treatment parallel-arm study in healthy male volunteers from Zimbabwe. Each group received itraconazole (ITZ; 100 mg once daily; *n* = 10), fluconazole (FKZ; 50 mg once daily; *n* = 9), or alprazolam (APZ; 1 mg once daily; *n* = 8) orally. Midazolam (MDZ), dosed orally and intravenously, was used as a comparator to validate the exploratory measures of CYP3A activity and the effects of known inhibitors. Urinary metabolic ratios of 1β-OH-DCA/ToDCA before and after CYP3A inhibitor treatment showed a similar magnitude of inhibitory effects of the three treatments as that measured by oral MDZ clearance. The maximum inhibition effect of a 75% reduction in the 1β-OH-DCA/ToDCA ratio compared to the baseline was achieved in the ITZ group following six once-daily doses of 100 mg. The correlations of the two markers for CYP3A inhibitor treatment were significant (r_s_ = 0.53, *p* < 0.01). The half-life of urinary endogenous 1β-OH-DCA/ToDCA was estimated as four days. These results suggested that 1β-OH-DCA/ToDCA in spot urine is a promising convenient, non-invasive, sensitive, and relatively quickly responsive endogenous biomarker that can be used for CYP3A inhibition-based drug–drug interaction in clinical studies.

## 1. Introduction

The evaluation of the likelihood and magnitude of drug–drug interaction remains an integral part of drug candidate safety assessments during drug discovery, development, and regulatory review. Probe drugs that are exclusively metabolised by drug-metabolising enzymes of interest are often used in these evaluations. This, however, involves the administration of probe drugs followed by invasive sampling. Additionally, it might pose a risk for adverse effects and difficulty in conducting clinical drug–drug interaction (DDI) studies in patient populations. There is therefore a need for the development of sensitive endogenous biomarkers, especially urinary biomarkers, as an alternative phenotyping method to overcome this challenge. Some endogenous compounds have been demonstrated to have potential as markers that mirror the interindividual variation of enzyme activities in drug metabolism [1,2,3]. The identification and validation of such endogenous markers could be useful in the evaluation of new chemical entities for the risk of drug-metabolising enzyme inhibition or induction-based DDI.

The vast majority of clinical DDI studies have investigated the effect of drugs on the CYP3A family because they are collectively the most abundant of all human CYP isoforms, with CYP3A4 accounting for 30–40% in the liver and intestine [4,5]. The CYP3A family is involved in the metabolic clearance of more than 27–30% of all drugs [4,6] and is a major source of variability in drug response. This makes the family of great interest in clinical DDI investigations [7]. Accordingly, it is important to accurately estimate the impact of the administered drug on CYP3A activity in the early phase of drug development. Several drug-metabolising enzyme probes have been utilised [8]. Midazolam (MDZ) is a sensitive in vivo drug probe that is commonly used to assess the inhibition and induction of CYP3A. Studies using this probe drug, however, require hospital visits where participants are required to provide timed and invasive blood sample collections. A sensitive biomarker, preferably measurable in urine spot samples with a fast response to changes in CYP3A activity would be of great benefit for the assessment of CYP3A DDIs and phenotyping in vivo.

There have been encouraging results when CYP3A endogenous biomarkers have been used to capture potential DDIs during early drug development, as well as to guide the design of clinical studies [1]. Cortisol, cortisone, and dehydroepiandrosterone (DHEA) are extensively metabolised by CYP3A4, with urinary 6β-hydroxycortisol/cortisol and 6β-hydroxycortisone/cortisone ratios being shown to be useful CYP3A activity predictors [1,9,10,11,12]. The plasma concentration of 4β-hydroxycholesterol (4βHC) has also been suggested as a potential endogenous CYP3A biomarker [13]. However, there are shortcomings in the implementation of these markers in clinical settings, including a lack of specificity in the case of urinary 6β-hydroxycortisol/cortisol [9], a lack of quick response due to the long half-life of 4βHC [14], intraindividual variation, and diurnal rhythm.

The potential usefulness of deoxycholic acid (DCA) to 1β-hydroxy-deoxycholic acid (1β-OH-DCA) as an endogenous urinary biomarker was evaluated in our previous studies in vitro and in vivo using spot urine from a CYP3A-inducer, carbamazepine-treated patient [15]. The study demonstrated that the metabolic reaction of DCA to 1β-OH-DCA by CYP3A was selective and sensitive, as was well-reflected in the studied spot urine samples. This preliminary result in one individual, however, needs to be evaluated in a large size of clinical samples to establish its variability over time and interindividual differences. This study aimed to generate data concerning urinary 1β-OH-DCA/total DCA (1β-OH-DCA/ToDCA) as a marker of CYP3A activity in healthy subjects and its response to potent, intermediate, and weak inhibitors of CYP3A. The usefulness of this potential biomarker to reliably detect and estimate levels of change in CYP3A4 activity was evaluated using itraconazole (ITZ), fluconazole (FKZ), and alprazolam (APZ) as the diagnostically potent, intermediate, and weak inhibitors, respectively [16]. MDZ was administered and used as a model substrate of CYP3A activity for comparison.

## 2. Materials and Methods

### 2.1. Chemicals

Investigational materials were obtained from various sources as follows: APZ tablets (1 mg of Xanax) and FKZ tablets (50 mg of Diflucan) were obtained from Pfizer (Pfizer, NY, USA). MDZ (5 mg/5 mL Domicum ampules) was obtained from Roche (Roche, Basel, Switzerland). MDZ (2.5 mg/0.5 mL buccolam) was obtained from Shire Pharmaceuticals (Shire Pharmaceuticals, London, United Kingdom), and ITZ tablets (200 mg of Sporanox) were obtained from Janssen-Cilag (Janssen-Cilag, NJ, USA). 

MDZ, 1′-OH-MDZ, DCA, diazepam, activated charcoal (DARCO^®^ G-60, 100 mesh powder), and TRIS hydrochloride were obtained from Sigma Aldrich (GmbH, Germany). DCA-2,2,4,4-d4 (Lot No. M403P52, 99 atom %D, (D4)-DCA) was purchased from CDN isotopes, Quebec, Canada. References of 1β-OH-DCA, 2,2,4,4-d4-1β-OH-DCA ((D4)-1β-OH-DCA), DCA-3-sulfate (DCA-3-S), glycine and taurine conjugated DCA, and 1β-OH-DCA (G-DCA, T-DCA, G-1β-OH-DCA, and T-1β-OH-DCA) were obtained from Isotope Chemistry, AstraZeneca, Gothenburg, Sweden. Chenodeoxycholic acid-24-acyl-β-D-glucuronide (CDCA-24-G) was purchased from Toronto Research Chemicals (Toronto, Canada). Choloylglycine hydrolase (from *Clostridium perfringens*), β-glucuronidase/arylsulfatase (from *Helix pomatia*), and sulfatase (from *Helix pomatia*, Type H-1) were purchased from Sigma-Aldrich (Dorset, UK). All other reagents were of analytical grade.

### 2.2. Study Population and Design

Fifty healthy male volunteers had their full medical history taken and a physical examination performed. Laboratory tests for Hepatitis B, Hepatitis C, and HIV were performed. All subjects were screened for abnormal laboratory values (alanine transaminase (ALT), γ-glutamate transferase (γ-GT), aspartate transaminase (AST), creatinine, sodium, chloride, potassium, urea, haematocrit, haemoglobin, and white cell count). The study’s inclusion criteria included male status, an age range of 18–30 years, a doctor’s pass for general health through physical examination, blood pressure, and biochemical measures within standard ranges for a healthy individual. The study excluded individuals with a history of drug or alcohol abuse, smokers, and those on any herbal or prescription medication. 

All participants in this trial gave their written informed consent to participate in this study. Appropriate study approvals were obtained from the Medical Research Council of Zimbabwe and the Medicines Control Authority of Zimbabwe. The participants were identified according to the inclusion and exclusion criteria set for the study. A total of 30 young male volunteers who met the study inclusion criteria were enrolled in the study. One participant withdrew consent during the follow-up period. 

The clinical trial was an open-label, three parallel-arm study to evaluate the effects of a strong (100 mg of ITZ once-daily (od) for 6 days) [17,18], intermediate (50 mg of FKZ od for 14 days) [17,19], and weak inhibitor (1 mg of APZ od for 9 days) [16] on the CYP3A4-mediated formation of 1β-OH-DCA, MDZ clearance, and the formation of 1-OH-MDZ. ITZ tablets (200 mg) were split equally in two, and 100 mg were given to subjects. 

The trials were conducted following the ethical principles outlined in the Declaration of Helsinki and the International Conference on Harmonization of Good Clinical Practice. Written informed consent was obtained from all subjects before any study-related procedures. Ethical approval was obtained from the Medical Research Council of Zimbabwe (MRCZ/A/2143).

**Figure 1:**
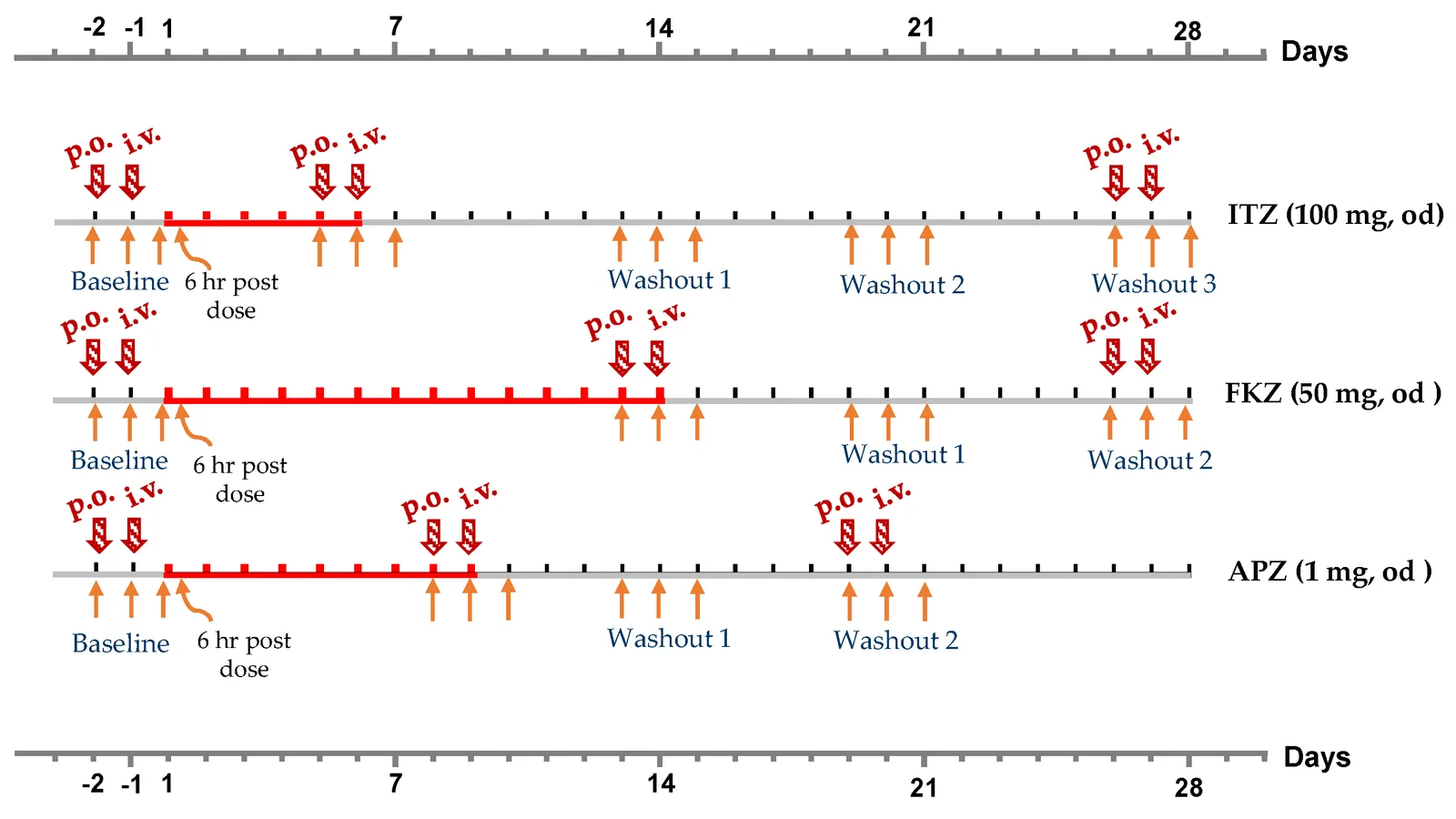
Study design. The red marked date indicates the ITZ/FKZ/APZ daily administration. The striped arrow indicates the MDZ AUC plasma sampling. The orange arrow indicates spot urine sampling, which was collected the morning before drug administration except for the 6 h post-dose urine collection on Day 1. (p.o.—oral administration; i.v.—intravenous administration; od—once-daily).

The study design, including the sampling of different measurements, is presented in Figure 1. The study sample size for each arm (*n* = 10) was calculated using Schumann’s two, one-sided *t*-test procedure to determine the minimum sample size (*n*) accounting for 20% dropout. MDZ acted as a nested control in the evaluation of the endogenous urinary markers, and it was administered both orally (1.5 mg) and intravenously (i.v.; 1.0 mg). Participants were all treated the same way for the baseline and randomly split into 3 arms of 8–10 individuals each to evaluate the effect of the inhibitors. Blood sampling to determine MDZ pharmacokinetics was conducted at time points 0.25, 0.5, 0.75, 1, 1.5, 2, 3, 4, 6, 8, 12, and 16 h. Spot urine biomarker sampling was conducted at 0 hr (baseline), 6 hr and 24 hr post ITZ/FKZ/APZ dosing. MDZ pharmacokinetic (PK) profiling and biomarker sampling was also conducted after the washout period and at the end of the study to determine whether enzyme activity returned to baseline levels. The subjects were their control. Treatment, washout and end of study values were compared with values at baseline. 

### 2.3. Quantification of MDZ and 1′-OH-MDZ in Plasma 

Plasma concentrations of MDZ and its metabolite 1′-OH-MDZ were analysed using a validated LC-MS/MS method. Plasma samples were extracted using protein precipitation, where 300 µL of acetonitrile (ACN) containing 500 ng/mL diazepam as an internal standard (IS) was added to 100 µL of plasma. The mixture was then vortexed for 10 min, followed by centrifugation at 10,000× *g* for 5 min at a temperature of 4 °C. The supernatant was collected, and 5 µL were injected into the LC-MS/MS for analysis. 

Chromatographic separation was achieved on a Phenomenex Synergi™ 4 µm Polar-RP 80 Å, LC Column 150 × 2 mm. The mobile phase consisted of 10 mM ammonium formate in 5% methanol (MeOH) (A) and ACN (B). Analytes were eluted using a gradient programmed as follows: 30% B (0–0.5 min), 60% B (0.5–1.5 min), 90% B (1.5–4.3 min), and 30% B (4.4–5.5 min) at a flow rate of 0.25 mL/min. The column temperature was maintained at 40 °C. MDZ and 1′-OH-MDZ were detected on a Sciex 3200 mass spectrometer with a turbo ion spray interphase operated in positive ion mode at 5500 V, a temperature of 5000 °C; GS1 and GS2 at 50 and 60 psi, respectively; and a curtain gas flow of 25 psi. The multiple reaction monitoring mode for specific precursor/product ion was used to detect MDZ (*m/z* 326.7 > 244.1), 1′-OH-MDZ (*m/z* 342.7 > 324, 203, and 168.1), and diazepam (285 > 154). The quantification of the MDZ and 1′-OH-MDZ was performed using the Analyst software (version 1.2; Applied Biosystems). 

### 2.4. Quantification of 1β-OH-DCA and ToDCA in Urine

Sample preparations for the analysis of 1β-OH-DCA and ToDCA in human urine were performed using a previously described method but without acid solvolysis [15,20]. In brief, an aliquot of 50 µL of a 0.1 M Tris HCL buffer (pH 5) containing a mixture of hydrolysis enzymes (100 U/mL choloylglycine hydrolase, 200 µL/mL β-glucuronidase/arylsulfatase, and 100 U/mL sulfatase) was added to 50 µL of urine, followed by adding 50 µL of an aqueous solution containing 1 µM of (D4)-DCA and (D4)-1β-OH-DCA as IS. The mixture was incubated overnight at 37 °C at a shaking speed of 500 rpm for enzymatic hydrolysis of glucuronides, sulfates, and amino acid conjugates. The reaction mixture was then quenched with a 300 µL ACN-MeOH (1:1, *v/v*) solution followed by centrifugation at 4000× *g*, 4 °C for 20 min. The supernatant was separated and diluted with an equal volume of water for ultra-high-performance liquid chromatography-high resolution mass spectrometry (UPLC-HRMS) analysis.

Calibration standards were constructed in stripped-pooled human urine using the method described by Bathena et al. [21]. To 5 mL of blank urine, 500 mg of activated charcoal were added and shaken in a horizontal position at 300 rpm at 30 °C for 2 h. The content was then centrifuged (4000× *g* at 4 °C) for 20 min, and the supernatant was filtered using a 0.22 µm nylon filter. Serial concentrations of a mixture of DCA, DCA-3-S, and 1β-OH-DCA were spiked into the striped urine to prepare calibration curves in the range of 7–5000 nM of DCA and DCA-3-S and 2–1600 nM of 1β-OH-DCA. The calibration standards were then subjected to the sample preparation described above but without overnight incubation with enzymes. Instead, the calibration urine mixtures were placed on wet ice and immediately quenched by an organic solvent, followed by the rest of the steps to prepare samples for UPLC-HRMS analysis.

References containing 10 µM G-DCA, T-DCA, G-1β-OH-DCA, T-1β-OH-DCA, CDCA-24-G, and DCA-3-S were also prepared in stripped urine and subjected to enzymatic hydrolysis to monitor the efficiency of enzymatic deconjugation. As a result, the enzymatic deconjugation was complete for amide and glucuronide conjugates. However, DCA-3-S was rather stable with less than 30% hydrolysed (Data not shown). Therefore, the concentrations of DCA-3-S were also determined in urine and combined with free DCA to represent the total concentration of DCA, i.e., ToDCA.

A Synapt G2-Si Q-TOF mass spectrometer coupled with an ACQUITY UPLC system (Waters Corp.) was used to separate, identify, and quantify 1β-OH-DCA, DCA, and DCA-3-S. UPLC separations were performed on an Acquity UPLC BEH C18 column (2.1 × 100 mm, 1.7 μm; Waters, Milford, MA). Mobile phase A was a 0.1% formic acid aqueous solution, and mobile phase B was ACN. The initial mobile phase was 90:10 A–B and was transitioned via a linear gradient to 25:75 A–B over 5 min, followed by 1 min of a washing period at 10:90 A–B for 1 min before returning to the initial condition. The flow rate was 0.5 mL/min, and the total run time was 7 min. The column oven and autosampler were set at 45 and 8 °C, respectively. 

The UPLC eluent was introduced into the Synapt G2-Si Q-TOF mass spectrometer with an electrospray (ESI) interface. Full-scan mass spectra were acquired under the negative ESI modes. Specific mass spectrometric source conditions were as follows: a capillary voltage of 0.5 kV, sample cone voltage of 40 V, desolvation temperature of 550 ºC, and a source temperature of 150 °C. All MS data were acquired using a low collision voltage (4 eV) to generate precursor ion spectra and chromatograms. All data were acquired in centroid mode with a mass range of *m/z* 100–1200. Leucine-enkephalin was used as an internal calibrant for accurate mass measurements. MassLynx (version 4.1, Waters Corp., Taunton, MA, USA) was used to control the system, for data acquisition, and for analysis. TargetLynx (Waters Corp.) was used to process the data for the quantification of 1β-OH-DCA, DCA, and DCA-3-S. Extracted ion chromatograms (XICs) of DCA (*m/z* of 391.285; retention time t_R_ of 4.53 min) and its IS (D4)-DCA (*m/z* of 395.310; t_R_ of 4.53 min), as well as 1β-OH-DCA (*m/z* of 407.280; t_R_ of 3.15 min) and its IS (D4)-1β-OH-DCA (*m/z* of 411.305; t_R_ of 3.15 min), were generated. XICs of DCA-3-S (*m/z* of 471.242; t_R_ of 3.98 min) were also extracted for quantification, with (D4)-DCA being used as its IS. A mass tolerance window of 10 mDa was applied for all XIC for quantification analysis. 

The concentrations of 1β-OH-DCA, DCA, and DCA-3-S were calculated based on the peak area ratio of each compound to their respective IS and compared with the calibration curves. The urinary metabolite ratios (UMRs) of concentrations of 1β-OH-DCA to total DCA (i.e., the sum of determined DCA and DCA-3-S) were therefore calculated for spot urine samples in each study arm to evaluate its potential usability as a biomarker for CYP3A activity.

### 2.5. Statistical Analysis

Standard non-compartmental pharmacokinetic (PK) analysis was performed with the WinNolin program, version 4.1 (Pharsight Corp, Mountain View, CA, USA), using the linear up logarithmic down trapezoidal method. The plasma parameters that were assessed and used for the comparison with the urinary biomarker were MDZ clearance (MDZ CL) and the area under the plasma concentration-time curve (AUC) from zero to infinity (AUC_0–inf_) of MDZ. More PK parameters were evaluated for MDZ and 1′-OH-MDZ. All MDZ PK data assessment can be found in the Supplemental Materials. 

To test for statistically significant differences of the 1β-OH-DCA-based UMR before and after the treatment of ITZ/FKZ/APZ, the UMRs were log-transformed. The difference between log-transformed treatment/baseline was calculated pairwise for each subject, as well as for their 90% confidence intervals (CIs). These differences and CIs were anti-log-transformed back for reporting purposes. The same analysis was performed for MDZ clearance and AUC_0-inf_ before and after treatment for comparison. Furthermore, the statistically significant differences of the two markers, i.e., 1β-OH-DCA/ToDCA, UMR, and MDZ clearance, to reflect CYP3A activity were also analysed. The difference between the log-transformation of 1β-OH-DCA-based UMR_Treatment_/UMR_Baseline_ and MDZ-based CL_Treatment_/CL_Baseline_ were calculated. The mean differences and 90% CIs for the differences were exponentiated to provide point estimates of the ratio of geometric means (1β-OH-DCA/ToDCA UMR: MDZ CL) and 90% CIs for the ratios. 

The correlation of the influence of ITZ/FKZ/APZ treatment on CYP3A activity was measured using log-transformed treatment/baseline ratios derived by 1β-OH-DCA/ToDCA UMR, and MDZ clearances were evaluated using Spearman’s rank correlation coefficients with a two-sided *p*-value (α = 0.05). All correlation, linear regression, and descriptive statistics analysis were performed using GraphPad Prism 9 (GraphPad Software, San Diego, CA, USA). *p* ≤ 0.05 was considered statistically significant.

## 3. Results

### 3.1. Baseline Characteristics of the Study Participants

The baseline demographics of all male participants were balanced between the study arms (Table 1) with no statistically significant differences according to the Kruskal–Wallis test. Median age, weight, and BMI were 23 years (χ^2^ = 2.44; *p* = 0.29), 61.6 kg (χ^2^ = 2.33; *p* = 0.31), and 21.2 kg/m^2^ (χ^2^ = 3.38; *p* = 0.18), respectively. 

### 3.2. Time Course of CYP3A-Mediated 1β-Hydroxylation of DCA in Urine Following Repeated Administration of ITZ, FKZ, and APZ 

**Figure 2:**
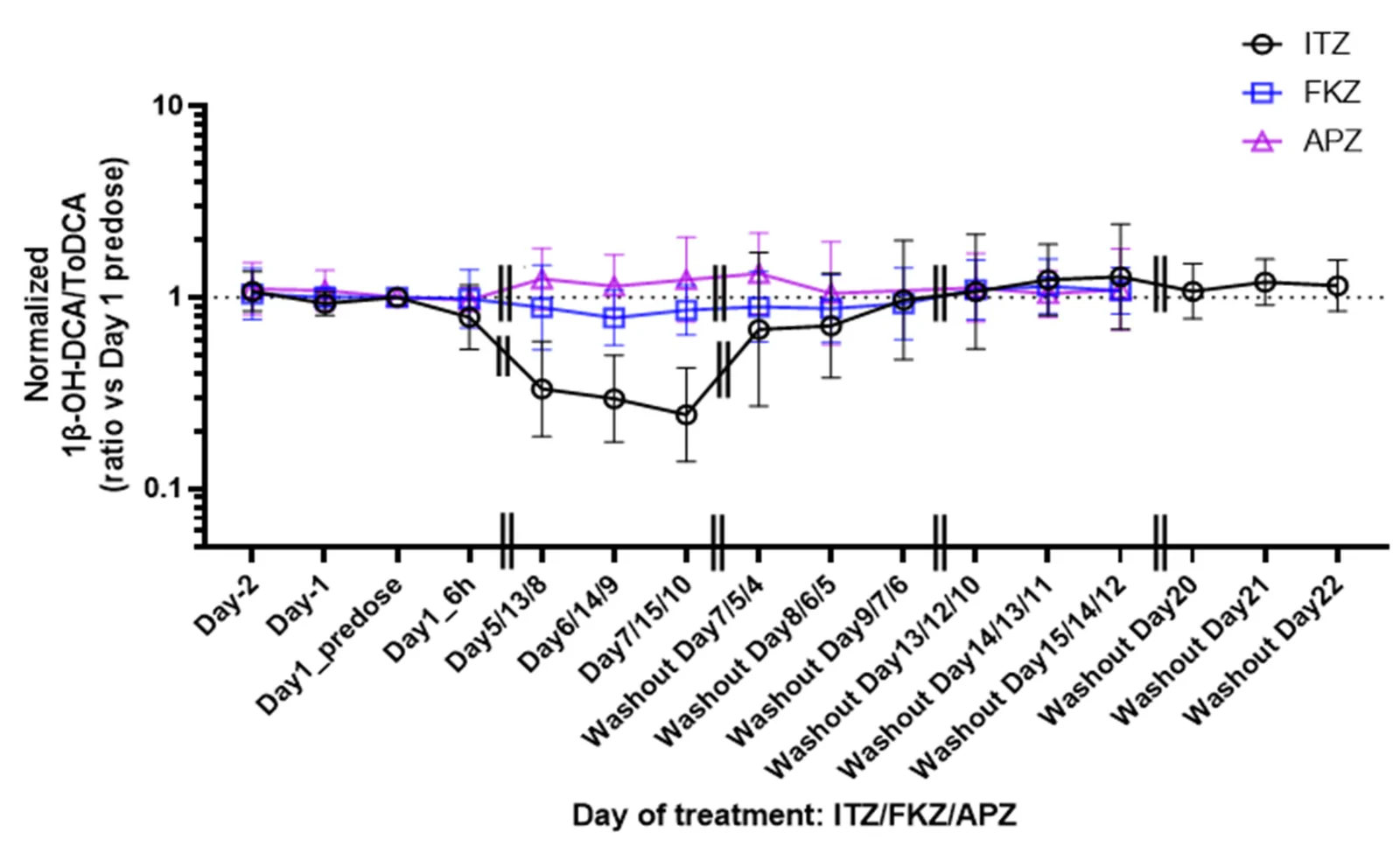
Plot of changes of the urinary metabolic ratio of 1β-OH-DCA/ToDCA from baseline in healthy male volunteers before, during, and after the repeated administration of ITZ (100 mg od; *n* = 10), FKZ (50 mg od; *n* = 9) and APZ (1 mg od; *n* = 8).

The time-course of the measured 1β-OH-DCA/ToDCA ratios were normalized using pre-dose values on Day 1. A decreased 1β-OH-DCA/ToDCA UMR from baseline was observed following ITZ treatment (100 mg) (Figure 2). The effect was lower in the FKZ (50 mg) group. No inhibition was observed in the APZ (1 mg) group. The ratios returned to baseline after the washout period. Following a single dose of ITZ, the ratio of 1β-OH-DCA/ToDCA decreased 21% from baseline in the 6 h post-dose urine. Following repeated once-daily doses of ITZ for four days, a significant 67% reduction in 1β-OH-DCA/ToDCA was observed and continued to decline to 75% after six, once-daily, 100 mg ITZ oral doses. The 1β-OH-DCA/ToDCA ratio was fully restored to baseline nine days after the completion of ITZ treatment. 

**Figure 3:**
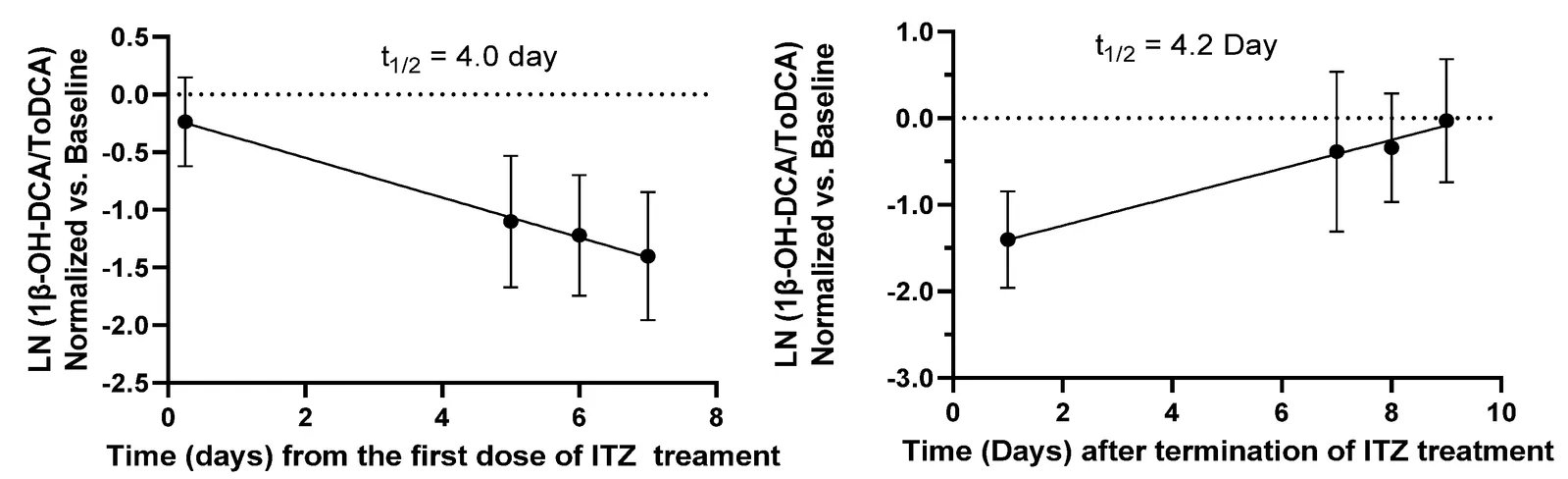
Elimination and recovery curves for urinary 1β-OH-DCA/ToDCA UMR in ten healthy volunteers treated with ITZ (100 mg od) for 6 days.

Based on the time course of 1β-OH-DCA/ToDCA following ITZ treatment, the half-life of urinary 1β-OH-DCA/ToDCA was estimated using the elimination curve following once-daily doses of ITZ and the recovery curve during the first washout period after the termination of ITZ treatment (Figure 3). Calculations of the elimination and the recovery rate using the existing data points resulted in a half-life of about four days.

### 3.3. Inhibition of CYP3A-Mediated 1β-Hydroxylation of DCA and MDZ Clearance 

MDZ was used as a nested control to compare and evaluate the effectiveness of the 1β-OH-DCA-based UMR biomarker. Calculated MDZ pharmacokinetic parameters before and after inhibitor administration are summarized in the Supplementary Materials (Figure S1 and Table S1, Table S2 and Table S3). The data obtained from 1′-OH-MDZ showed similar trend to that of MDZ clearance. Because MDZ exposure is the most accepted marker for CYP3A DDI assessment, we decided to choose MDZ clearance for comparison and correlation analysis with the 1β-OH-DCA/ToDCA UMR, and no further comparison with 1′-OH-MDZ data is reported here.

**Figure 4:**
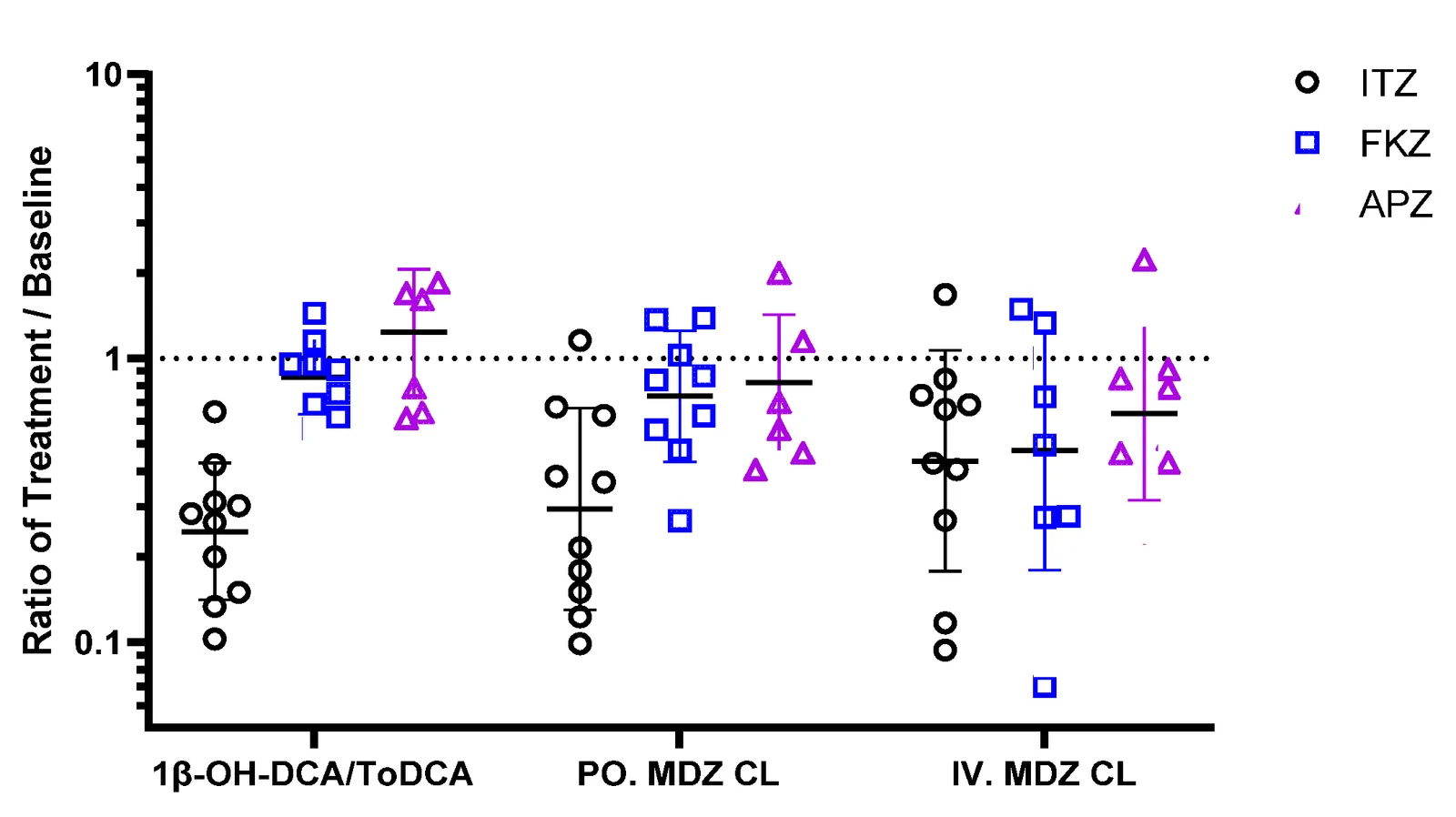
Comparison of the changes of 1β-OH-DCA/ToDCA UMR, oral, and i.v. clearance of MDZ from baseline following the repeated administration of ITZ (100 mg od for 6 days), FKZ (50 mg od for 14 days), and APZ (1 mg od for 9 days).

**Figure 5:**
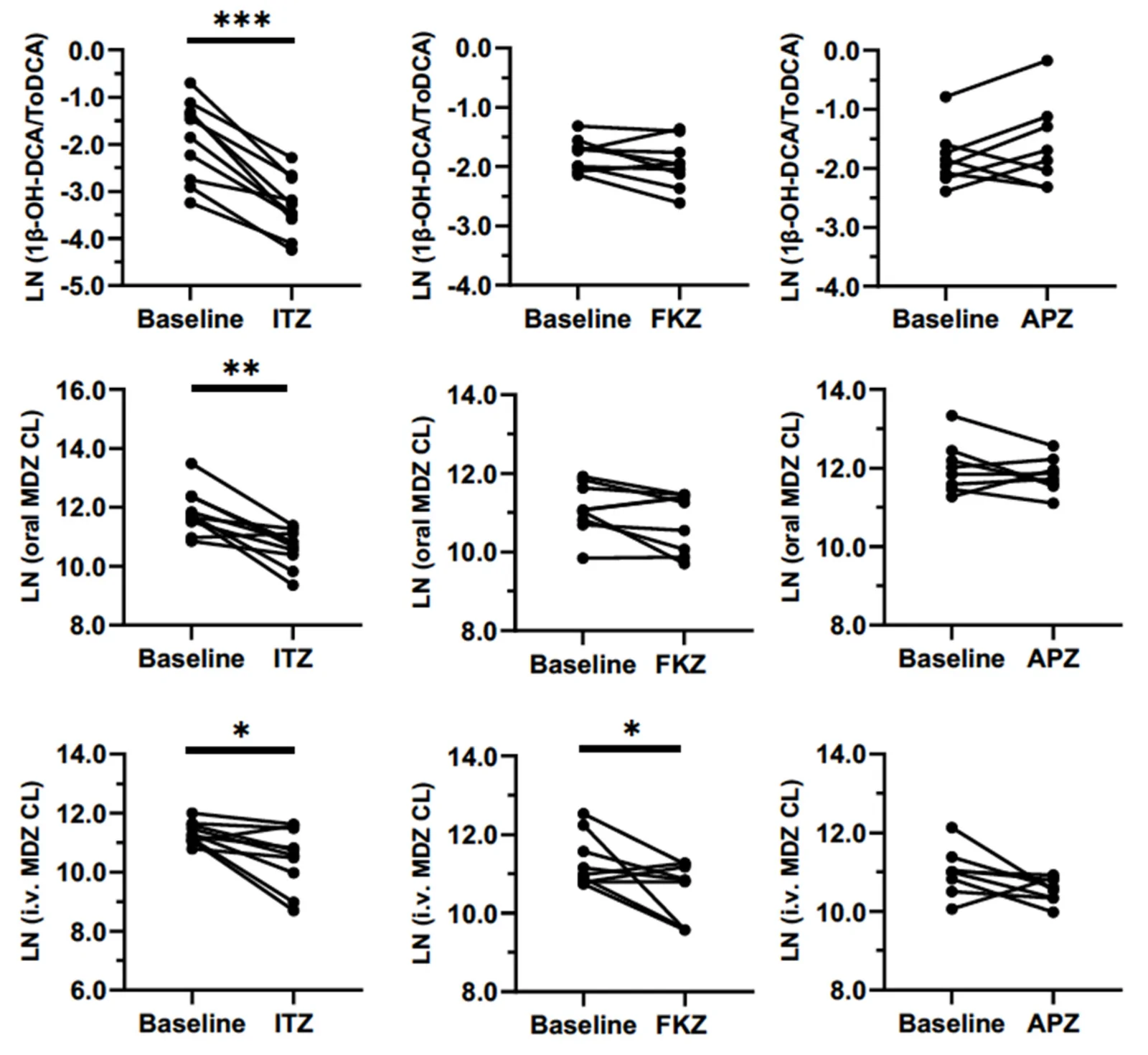
Individual urinary 1β-OH-DCA/ToDCA UMR (upper panel), oral (middle panel), and i.v. (bottom panel) MDZ clearance (L/h) at baseline and after the repeated administration of ITZ (100 mg od for 6 days), FKZ (50 mg od for 14 days), and APZ (1 mg od for 9 days). * *p* < 0.05; ** *p* < 0.01; *** *p* < 0.001.

The impact of ITZ, FKZ, and APZ on 1β-OH-DCA/ToDCA UMR and MDZ clearance following repeated administration is shown in Table 2 and Figure 4 and Figure 5. The 1β-OH-DCA/ToDCA ratios obtained in the pre-dose spot urine samples on Day 1 were used as the baseline and compared with that obtained at 24 h after the last inhibitor treatment, as well as the last day of washout period.

As expected, the greatest effect was obtained in the ITZ group when evaluated using different marker measurements. Oral MDZ CL significantly decreased (70%; *p* < 0.01) from baseline, with a 3.4-fold increase in AUC_0-inf_ observed after the repeated administration of ITZ (100 mg od) for six days. Similar effects were observed in the urinary biomarker activity, evaluated as 1β-OH-DCA/ToDCA UMR, with a significant reduction (*p* < 0.001) signifying the reduced activity of the CYP3A enzyme (Figure 4). However, 1β-OH-DCA/ToDCA UMR appeared to be less inhibited following FKZ (50 mg) and APZ (1 mg) group administration, with less significant increases of oral (mean of 1.2–1.4-fold) and i.v. (mean of 1.5–2.1-fold) MDZ AUC observed. The 1β-OH-DCA/ToDCA UMR and oral MDZ clearance showed non-significant decreases of 14% and 26% reduction, respectively, in the FKZ group compared to the baseline. 

The ratio of treatment/baseline values of UMR divided by the corresponding values calculated from MDZ clearance with/without inhibitor treatment were analysed to explore the potential equivalence of these two markers (Table 2 and Figure 5). As a result, the two markers showed no statistically significant differences (*p* > 0.05) in terms of mirroring the CYP3A activity changes, as measured by 1β-OH-DCA/ToDCA UMR and oral MDZ clearance with/without inhibitors. In the APZ group, although no differences were shown before and after the APZ doses measured by either 1β-OH-DCA/ToDCA UMR or MDZ clearance, a significant difference (*p* < 0.05) was observed between the two measurements by 1β-OH-DCA/ToDCA UMR and i.v. MDZ clearance. The data also suggested less interindividual variability for the urinary marker compared to MDZ clearance, as indicated by the spread of data around the median (Figure 5).

### 3.4. Correlation of 1β-OH-DCA/ToDCA Ratio and MDZ Plasma Clearance

**Figure 6:**
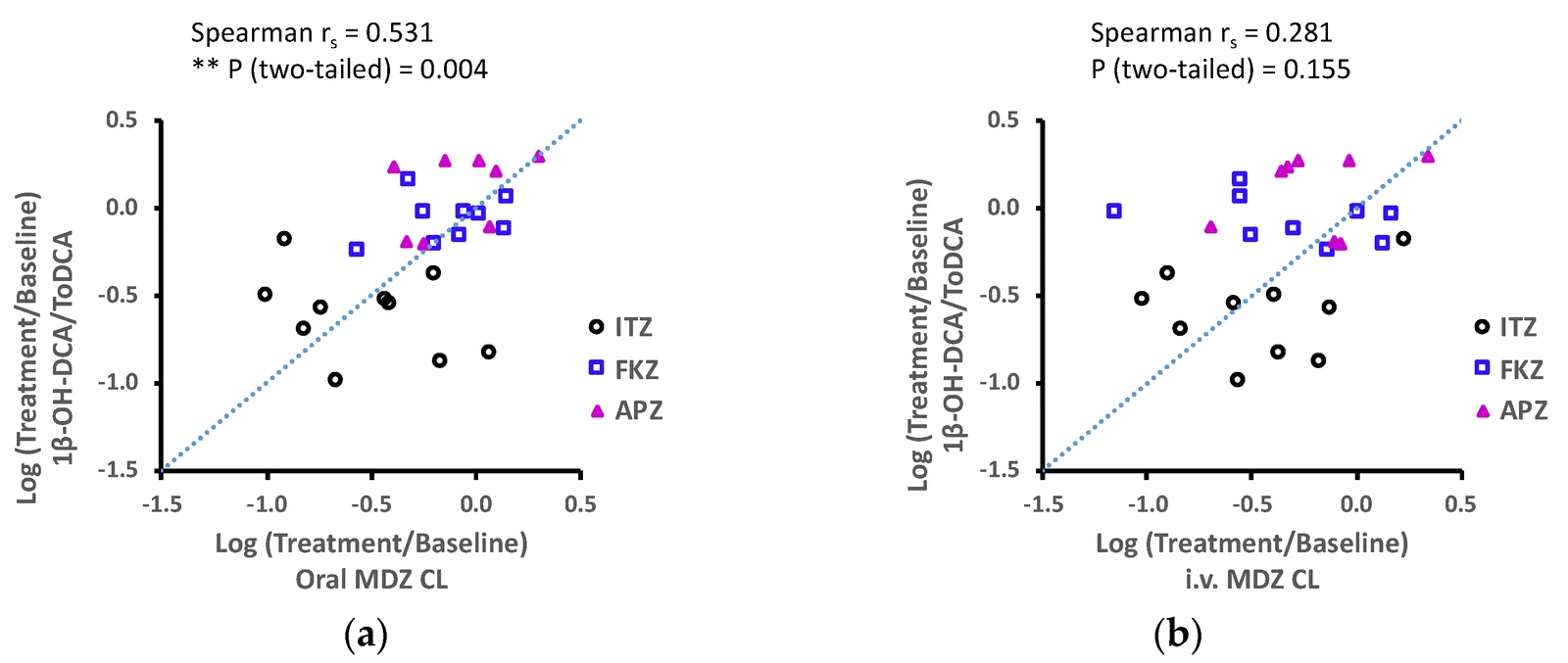
Spearman correlation between changes of the urinary metabolic ratio of 1β-OH-DCA/ToDCA and orally (**a**) and i.v. (**b**) administered MDZ clearance before (baseline) and after the repeated administration of ITZ (100 mg od for 6 days), FKZ (50 mg od for 14 days), and APZ (1 mg od for 9 days).

The correlations between the impact of 1β-OH-DCA/ToDCA levels and MDZ clearance before and after CYP3A inhibitor treatment were estimated using Spearman’s rank correlation coefficients. As shown in Figure 6, the differences of 1β-OH-DCA/ToDCA UMR correlated significantly with oral MDZ clearance (*r*_s_ = 0.53; *p* = 0.004). The correlation was, however, weak with i.v. MDZ clearance (*r*_s_ = 0.28; *p* = 0.155). The inhibition of the UMR agreed with that of MDZ clearance, as indicated by the unity line (Figure 6). The regression slope to unity was used as an indication of accuracy, i.e., the extent of inhibition was the same with the two methods. 

## 4. Discussion

This study was designed to evaluate the usefulness of the metabolite ratio of 1β-OH-DCA/ToDCA as a biomarker for CYP3A activity in the assessment of inhibition-based drug–drug interactions. MDZ, a well-known probe substrate for evaluating CYP3A activity, was used as a nested control [22] in an open-label, three parallel-arm study utilizing healthy male participants. APZ (1 mg), FKZ (50 mg) and ITZ (100 mg) were used as diagnostic weak, moderate and potent inhibitor respectively. The inhibitors were administered once-daily at the clinically relevant doses until they reached a steady-state to evaluate the inhibition of CYP3A. The 1β-OH-DCA was previously demonstrated to be a potential endogenous urinary marker, as it was selectively metabolized by CYP3A and CYP46A1 [15]. Metabolism by the latter is not of concern because CYP46A1 is located in the brain where the levels of bile acids are very low [23]. Spot urine samples were collected in this study, and the concentration of CYP3A-mediated 1β-OH-DCA formation was normalized by the total concentration of DCA in a paired fashion for each individual. The changes of the urinary ratios of 1β-OH-DCA/ToDCA before and after ITZ/FKZ/APZ treatment were analysed and compared with the associated individual MDZ CL. 

The time course of 1β-OH-DCA/ToDCA UMR changes before and after ITZ treatment and following the washout period (Figure 2) indicated a gradual decrease of 1β-OH-DCA/ToDCA UMR and its recovery. A limited immediate inhibition (21% reduction) of 1β-OH-DCA/ToDCA after a single ITZ dose was observed, which was consistent with a 22% decrease reported by Magliocco et al. following the first day of voriconazole and fluvoxamine doses [24]. The response of endogenous biomarkers to perpetrators depends on their baseline levels, their formation, and their elimination, unlike probe drugs whose responses rely on absorption and elimination. This low immediate response of 1β-OH-DCA/ToDCA UMR to inhibitors can be explained by the half-life of the endogenous biomarker, which was estimated to be four days based on the elimination and recovery curves at the inhibition phase or after the termination of ITZ (Figure 3). The low response could also be explained by the duration of inhibition. It would require time and/or enough inhibitor concentrations to eliminate these baseline levels before any significant inhibitory effects could be observed. If this is to be equated to the elimination half-life of drugs, it would require at least four times the elimination half-life of the endogenous metabolite to capture maximal inhibition [25]. It is therefore unlikely to capture CYP3A inhibition 6 h after inhibitor inhibition, especially for the moderate and weak inhibitors. Accordingly, it would be crucial to evaluate if the biomarker changes are reversible. We observed a full reversal of the inhibitory effects nine days after the termination of the administration in the ITZ arm. 

Sensitivity with weak and moderate inhibitors is one of the most important attributes of a biomarker. In this study, clinically relevant doses of APZ, FKZ, and ITZ were administered once-daily until they reached a steady-state to evaluate the inhibition of CYP3A. We expected the biomarker to be sensitive enough to differentiate between strong, moderate, and weak inhibitors. This sensitivity was very clear for 1β-OH-DCA/ToDCA UMR and oral MDZ clearance after the inhibitors reached a steady-state (Figure 5). The maximum inhibition of 1β-OH-DCA/ToDCA UMR was observed with a 75% reduction from baseline following ITZ repeated doses (100 mg od for 6 days), which was of the same magnitude as the inhibition of oral MDZ clearance (70%). Correspondingly, a 3.4-fold increase in oral MDZ AUC was detected. This result was in good agreement with the reported 85% reduced oral MDZ clearance and a 6.6-fold increase in AUC following ITZ (200 mg od for 6 days) treatment [17]. This large inhibition window and relatively short half-life are promising features making this endogenous marker suitable for its use in clinical studies. Furthermore, a recent study reported the modulation of 1β-OH-DCA/ToDCA UMR with an induction ratio of 11.4 following rifampicin intake in humans [24]. The same study also indicated the potential use of 1β-OH-DCA/ToDCA UMR in inhibition studies. It is worth noting that when the inhibition/induction effect approaches the maximum limit of the dynamic response of marker reactions, the changes of marker response to enzyme activity stop being linear. Further studies may be needed to explore the 1β-OH-DCA/ToDCA UMR response to strong CYP3A inhibition, which could cause a more-than 5-fold AUC increase of CYP index substrate, to understand the maximum inhibition of this 1β-OH-DCA/ToDCA marker and therefore its resolving power toward strong CYP3A inhibitions.

FKZ (50 mg) and APZ (1 mg) were dosed in the other two study arms to generate different magnitudes of CYP3A inhibition than ITZ. FKZ resulted in a non-significant 26% reduction in oral MDZ CL and a 14% reduction in the 1β-OH-DCA/DCA ratio compared to the baseline (Table 2). One study reported a 2–3-fold increase of MDZ AUC following 400 mg of FKZ ingestion [26], which agreed with the 1.4-fold increase of oral MDZ AUC at the 50 mg of FKZ dose given in this study. As a CYP3A substrate, APZ could compete with MDZ and cause a weak inhibition effect to a sensitive CYP3A index substrate [27]. APZ showed no significant effect on both MDZ PK parameters and 1β-OH-DCA/ToDCA UMR. 

DCA is a secondary bile acid within the bile acid metabolic network [28]. It is formed in the gut lumen by gut microbiota and transported via portal circulation to the liver, where it is available for metabolism and elimination. The relationships between the changes of 1β-OH-DCA/ToDCA UMR and oral MDZ CL after CYP3A inhibitor treatment versus baseline showed significant Spearman’s correlation coefficients (r_s_ = 0.53; *p* = 0.004), suggesting it is a potential metric to reflect CYP3A enzyme activities. However, a similar significant correlation was not observed between 1β-OH-DCA/ToDCA UMR and intravenous MDZ CL (Figure 6). There was no clear difference in the inhibitory effects of the three inhibitors for intravenously administered MDZ (Figure 5). This could explain the poor correlation between changes 1β-OH-DCA/ToDCA UMR and MDZ clearance (i.v) (Figure 6b). This also suggests the roles of FKZ and ITZ in inhibiting gut metabolism. There were significant differences between the inhibitory effects of ITZ on i.v vs. oral MDZ CL (Figure 5) and other PK parameters (Table 2 and Table S3). A similar observation was previously made [17]. This finding also strengthened the usability of 1β-OH-DCA/ToDCA as a CYP3A marker in oral drug DDI investigations, especially in implying the possible involvement of intestinal CYP3A-mediated DDIs.

CYP3A4 and CYP3A7 are responsible for the in vitro 1β-hydroxylation of both DCA and its conjugated forms (G-DCA and T-DCA) [29]. CYP3A5’s contribution is limited [24], and it has also been suggested that 1β-OH-DCA did not significantly undergo glucuronidation/sulfation. Instead, 1β-OH-DCA is mainly excreted as amidated forms in urine. We used the enzyme hydrolysis method to release free DCA and 1β-OH-DCA. The un-hydrolysed DCA-3-S concentrations were also measured and summed up with free DCA to obtain total DCA concentrations to capture the major DCA and 1β-OH-DCA derivative components to represent this 1β-OH-DCA/ToDCA UMR marker. However, it needs to be noted that most of the bile acid conjugates are also substrates of drug transporters [28,30,31], and their excretion in urine could be affected by transporter-involved DDIs. Additionally, the urinary ratio of 1β-OH-DCA/ToDCA could also be affected by renal function. Other factors such as liver toxicity may challenge the interpretation of biomarker levels. Further studies to evaluate these marker changes in different patient populations may help to understand the relevance of these factors and increase the clinical utility of this endogenous biomarker.

Baseline correlation analysis could be used to evaluate the usefulness of this urinary marker for CYP3A phenotyping. Our results did not show any correlation between 1β-OH-DCA/ToDCA UMR and MDZ CL. The extent of CYP3A4 reduction by the inhibitors was, however, found to be similar when comparing the 1β-OH-DCA/ToDCA UMR and MDZ CL. A previous study reported significant relationship between the log-transformed 1β-OH-DCA/ToDCA UMR over 8 h of interval collection and the log-transformed oral MDZ CL in 10 participants at baseline [24]. This could be an indication that spot urine sampling may not be suitable for this purpose. It should, however, be noted that the exposure of endogenous biomarkers depends on the rate of formation and elimination, whereas that of the exogenous marker depends on absorption and elimination. Therefore, a direct comparison of the two may underestimate the sensitivity of the biomarker. 

In conclusion, the results show that the urinary ratio of 1β-OH-DCA/ToDCA can be used as a non-invasive probe to detect CYP3A4 inhibition in vivo. It also reflects well on the induction of CYP3A activities [24]. This marker could be a very promising replacement to MDZ clinical studies for the inhibition or induction of CYP3A without probe drug treatment or invasive blood sampling for PK analyses. The use of an endogenous CYP3A marker has obvious advantages and may be of value, both during drug development and for monitoring CYP3A activity in patients on multiple medications. Additionally, the marker may be useful to guide the clinical plan and timing of clinical DDI studies. This would avoid the need to perform a dedicated DDI study, thereby reducing the cost and time spent domiciled in a clinical trial centre, as well as unnecessary drug exposure.

## Figures and Tables

**Table 1:** Baseline demographic data for all male participants.

	MDZ + ITZ	MDZ + FKZ	MDZ + APZ	All Participants
	(*n* = 10)	(*n* = 10)	(*n* = 9)	(*n* = 29)
Median age (range), y	23.0 (20.0–26.0)	23.0 (19.0–28.0)	21.0 (19.0–22.0)	23.0 (19.0–28.0)
Median weight (range), kg	61.0 (56.5–64.1)	66.9 (55.8–68.7)	58.0 (56.7–64.7)	61.6 (55.8–64.7)
Median BMI (range), kg/m^2^	20.4 (19.3–21.2)	22.1 (20.3–23.7)	21.26 (21.1–22.7)	21.2 (20.3–23.7)

BMI—body mass index; MDZ—midazolam; ITZ—itraconazole; FKZ—fluconazole; APZ—alprazolam.

**Table 2:** Endogenous and exogenous CYP3A markers in men before and after the repeated administration of ITZ/FKZ/APZ, including ratios of treatment/baseline.

	Baseline	Treatment	Last Washout	Treatment/Baseline (90%CI)	Ratio (90%CI)
ITZ (100 mg od)					
1β-OH-DCA/ToDCA	0.15 (0.04–0.50)	0.04 (0.01–0.10)	0.17 (0.05–0.45)	0.25 *** (0.18–0.34)	
Oral MDZ CL	126 (46.7–648.8)	37.1 (10.5–79.5)	80.5 (34.8–211.6)	0.30 ** (0.18–0.47)	0.83 (0.43–1.61)
Oral MDZ AUC_0-inf_	10.8 (2.1–29.1)	36.6 (17.1–129.0)	16.9 (6.4–38.9)	3.40 ** (2.11–5.45)	
i.v. MDZ CL	82.9 (48.6–162.1)	36.1 (6.0–111.4)	120 (62.2–262.5)	0.44 * (0.26–0.73)	0.56 (0.35–0.92)
i.v. MDZ AUC_0-inf_	12.1 (6.17–20.6)	27.7 (9.0–167.4)	9.8 (3.8–16.1)	2.30 * (1.37–3.86)	
FKZ (50 mg od)					
1β-OH-DCA/ToDCA	0.17 (0.12–0.27)	0.14 (0.07–0.26)	0.18 (0.10–0.42)	0.86 (0.71–1.03)	
Oral MDZ CL	59.8 (17.0–135.9)	44.0 (14.8–85.2)	104 (57.6–331.9)	0.74 (0.53–1.02)	1.17 (0.84–1.63
Oral MDZ AUC_0-inf_	22.7 (10.0–79.6)	30.8 (15.9–91.5)	13 (4.1–23.5)	1.36 (0.98–1.89)	
i.v. MDZ CL	80.7 (46.3–276.0)	38.3 (14.4–78.5)	95.1 (46.9–213.4)	0.47 * (0.260–0.866)	1.81 (0.90–3.63)
i.v. MDZ AUC_0-inf_	12.4 (3.6–21.6)	26.1 (12.7–69.47)	10.5 (4.7–21.3)	2.11 * (1.16–3.85)	
APZ (1 mg od)					
1β-OH-DCA/ToDCA	0.16 (0.09–0.46)	0.20 (0.10–0.84)	0.18 (0.08–0.32)	1.24 (0.88–1.74)	
Oral MDZ CL	59.1 (28.0–220.5)	48.6 (23.7–102.2)	63.6 (38.2–220.5)	0.82 (0.57–1.19)	1.51 (1.02–2.21)
Oral MDZ AUC_0-inf_	10.56 (6.04–18.49)	8.67 (5.12–14.69)	8.39 (3.81–18.47)	0.80 (0.54–1.18)	
i.v. MDZ CL	59.6 (23.3–185.9)	38.1 (21.5–55.5)	53.7 (24.8–81.8)	0.64 (0.400–1.023)	1.94 * (1.16–3.22)
i.v. MDZ AUC_0-inf_	16.78 (5.38–42.8)	26.25 (18.01–46.44)	18.6 (12.2–40.3)	1.56 (0.98–2.50)	

All data are presented as geometric means (range) with 90% confidence intervals (CIs). Ratio: (Treatment_UMR_/Baseline_UMR_)/(Treatment_MDZ CL/_Baseline_MDZ CL_); AUC (h*ng/mL): area under the plasma concentration–time curve; CL (L/h): clearance; DCA: deoxycholic acid; MDZ: midazolam; 1β-OH-DCA: 1β-hydroxy-deoxycholic acid; ToDCA: total deoxycholic acid; od: once daily. * *p* < 0.05; ** *p* < 0.01; *** *p* < 0.001.

## Data Availability

Data is contained within this article or Supplementary Material. Additional data that support the findings of this study are available on request from the corresponding author. The data are not publicly available because of privacy and ethical restrictions.

## Supplementary Materials

The following are available online at https://www.mdpi.com/article/10.3390/jpm11060457/s1, Data analysis of midazolam pharmacokinetics. Figure S1: Mean plasma concentrations of midazolam and 1′-hydroxymidazolam versus time following the administration of midazolam (1.5 mg p.o. or 1 mg i.v) alone and after chronic exposure with either APZ alprazolam (APZ), fluconazole (FKZ), or itraconazole (ITZ). Table S1: Effect of alprazolam (1 mg) on midazolam and 1′-hydroxymidazolam pharmacokinetics following single-dose administration (1.5 mg p.o or 1 mg i.v) in healthy participants (*n* = 10). Table S2: Effect of fluconazole (50mg) on midazolam and 1′-hydroxymidazolam pharmacokinetics following single-dose midazolam administration (1.5 mg p. o or 1 mg i.v) in healthy participants (*n* = 10). Table S3: Effect of itraconazole (100mg) on midazolam and 1′-hydroxymidazolam pharmacokinetics following single-dose administration (1.5 mg p. o or 1 mg i.v) in healthy participants (*n* = 9).
