# Correction: Li et al. Evaluation of 1β-Hydroxylation of Deoxycholic Acid as a Non-Invasive Urinary Biomarker of CYP3A Activity in the Assessment of Inhibition-Based Drug–Drug Interaction in Healthy Volunteers. *J. Pers. Med.* 2021, *11*, 457

**DOI:** 10.3390/jpm16070382

**Published:** 2026-07-17

**Authors:** Xue-Qing Li, Roslyn Stella Thelingwani, Leif Bertilsson, Ulf Diczfalusy, Tommy B. Andersson, Collen Masimirembwa

**Affiliations:** 1 Drug Metabolism and Pharmacokinetics, Research and Early Development, Cardiovascular, Renal and Metabolism, BioPharmaceuticals R&D, AstraZeneca, 431 83 Gothenburg, Sweden; xueqing.li@astrazeneca.com (X.-Q.L.); tommy.b.andersson@gmail.com (T.B.A.); 2 Department of Pharmaceutical Medicine, African Institute of Biomedical Science and Technology (AiBST), Block C, Wilkins Hospital Complex, Harare, Zimbabwe; rthelingwani@aibst.edu.zw; 3 Division of Clinical Pharmacology-C1:68, Department of Laboratory Medicine at Karolinska Institutet, Karolinska University Hospital, SE-141 86 Stockholm, Sweden; leif.bertilsson@ki.se; 4 Division of Clinical Chemistry, Department of Laboratory Medicine at Karolinska Institutet, Karolinska University Hospital (Huddinge), SE-141 86 Stockholm, Sweden; ulf.diczfalusy@ki.se

**Error in Table**

In the original publication [1], there was a transcription error of the oral midazolam (Table 2) and intravenous (Table S1) area under the curve (AUC) values. The corrected Table 2 and Table S1 appear below.

**Updating Conflicts of Interest Statement**

In the original publication [1], the Conflict of Interest declaration did not fully reflect the required disclosure of conflicts of interest. The corrected Conflict of Interest appear below.

**Conflicts of Interest:** Authors X.-Q.L. and T.B.A. were employed by the company AstraZeneca. Authors C.M. and R.S.T. were employed by the African Institute of Biomedical Science and Technology (AiBST). The authors declare that this study received funding from AstraZeneca and institutional infrastructure and operations support from AiBST. The funder had the following involvement with the study: the conception & design, the analysis, interpretation of data, the writing of this article.

The authors state that the scientific conclusions are unaffected. This correction was approved by the Academic Editor. The original publication has also been updated.

## Figures and Tables

**Table 2:** Endogenous and exogenous CYP3A markers in men before and after the repeated administration of ITZ/FKZ/APZ, including ratios of treatment/baseline.

	Baseline	Treatment	Last Washout	Treatment/Baseline (90%CI)	Ratio (90%CI)
ITZ (100 mg od)					
1β-OH-DCA/ToDCA	0.15 (0.04–0.50)	0.04 (0.01–0.10)	0.17 (0.05–0.45)	0.25 *** (0.18–0.34)	
Oral MDZ CL	126 (46.7–648.8)	37.1 (10.5–79.5)	80.5 (34.8–211.6)	0.30 ** (0.18–0.47)	0.83 (0.43–1.61)
Oral MDZ AUC_0-inf_	10.8 (2.1–29.1)	36.6 (17.1–129.0)	16.9 (6.4–38.9)	3.40 ** (2.11–5.45)	
i.v. MDZ CL	82.9 (48.6–162.1)	36.1 (6.0–111.4)	120 (62.2–262.5)	0.44 * (0.26–0.73)	0.56 (0.35–0.92)
i.v. MDZ AUC_0-inf_	12.1 (6.17–20.6)	27.7 (9.0–167.4)	9.8 (3.8–16.1)	2.30 * (1.37–3.86)	
FKZ (50 mg od)					
1β-OH-DCA/ToDCA	0.17 (0.12–0.27)	0.14 (0.07–0.26)	0.18 (0.10–0.42)	0.86 (0.71–1.03)	
Oral MDZ CL	59.8 (17.0–135.9)	44.0 (14.8–85.2)	104 (57.6–331.9)	0.74 (0.53–1.02)	1.17 (0.84–1.63
Oral MDZ AUC_0-inf_	22.7 (10.0–79.6)	30.8 (15.9–91.5)	13 (4.1–23.5)	1.36 (0.98–1.89)	
i.v. MDZ CL	80.7 (46.3–276.0)	38.3 (14.4–78.5)	95.1 (46.9–213.4)	0.47 * (0.260–0.866)	1.81 (0.90–3.63)
i.v. MDZ AUC_0-inf_	12.4 (3.6–21.6)	26.1 (12.7–69.47)	10.5 (4.7–21.3)	2.11 * (1.16–3.85)	
APZ (1 mg od)					
1β-OH-DCA/ToDCA	0.16 (0.09–0.46)	0.20 (0.10–0.84)	0.18 (0.08–0.32)	1.24 (0.88–1.74)	
Oral MDZ CL	59.1 (28.0–220.5)	48.6 (23.7–102.2)	63.6 (38.2–220.5)	0.82 (0.57–1.19)	1.51 (1.02–2.21)
Oral MDZ AUC_0-inf_	10.56 (6.04–18.49)	8.67 (5.12–14.69)	8.39 (3.81–18.47)	0.80 (0.54–1.18)	
i.v. MDZ CL	59.6 (23.3–185.9)	38.1 (21.5–55.5)	53.7 (24.8–81.8)	0.64 (0.400–1.023)	1.94 * (1.16–3.22)
i.v. MDZ AUC_0-inf_	16.78 (5.38–42.8)	26.25 (18.01–46.44)	18.6 (12.2–40.3)	1.56 (0.98–2.50)	

All data are presented as geometric means (range) with 90% confidence intervals (CIs). Ratio: (Treatment_UMR_/Baseline_UMR_)/(Treatment_MDZ CL/_Baseline_MDZ CL_); AUC (h*ng/mL): area under the plasma concentration–time curve; CL (L/h): clearance; DCA: deoxycholic acid; MDZ: midazolam; 1β-OH-DCA: 1β-hydroxy-deoxycholic acid; ToDCA: total deoxycholic acid; od: once daily. * *p* < 0.05; ** *p* < 0.01; *** *p* < 0.001.

**Table S1:** Effect of alprazolam (1 mg) on midazolam and 1′-hydroxymidazolam pharmacokinetics following single dose administration (1.5 mg p.o or 1 mg i.v) in healthy participants (*n* = 10).

Parameter		Geometric Mean Values (90% CI)		Geometric Mean Ratio (90% CI)	*p*-Value
		MDZ Alone	MDZ + APZ		
**MDZ**					
***i.v***	*AUC_0–∞_ (ng·h/mL)*	16.78 (11.16–25.24)	26.25 (21.18–32.53)	156 (102–240)	0.09
	*AUC_last_ (ng·h/mL)*	16.64 (11.04–25.07)	24.57 (18.67–32.22)	148 (93–234)	0.12
	*C_max_ (ng/mL)*	25.98 (12.75–52.93)	50.71 (44.81–55.95)	193 (99–377)	0.02
	*T_max_ (h)*	0.29 (0.22–0.37)	0.30 (0.24–0.37)		
	*T_1/2_ (h)*	2.06 (1.62–2.61)	3.73 (1.80–7.73)		
	*CL (L/h)*	59.88 (39.62–89.50)	38.09 (30.73–47.20)		
***p.o.***	*AUC_0–∞_* *(ng·h/mL)*	10.56 (6.04–18.49)	8.67 (5.12–14.69)	80 (54–118)	0.30
	*AUC_last_ (ng·h/mL)*	10.43 (7.84–13.86)	10.85 (3.92–6.45)	104 (71–153)	0.23
	*C_max_ (ng/mL)*	4.13 (3.33–5.11)	5.03 (3.92–6.45)	122 (89–166)	0.07
	*T_max_ (h)*	0.76 (0.60–0.95)	1.03 (0.81–1.31)		
	*T_1/2_ (h)*	1.63 (1.01–2.62)	1.47 (1.11–1.95)		
	*CL (L/h)*	137.34 (101–186)	136.46 (101–184)		
**1′-OH-MDZ**					
***i.v***	*AUC_0–∞_* *(ng·h/mL)*	4.49 (2.12–9.50)	2.25 (1.07–4.72)	49 (18–100)	0.03
	*AUC_last_ (ng·h/mL)*	2.54 (1.49–4.35)	1.81 (0.84–3.86)	71 (28–179)	0.26
	*C_max_ (ng/mL)*	1.24 (1.02–1.50)	1.06 (0.88–1.27)	85 (67–109)	0.09
	*T_max_ (h)*	0.81 (0.65–1.10)	0.77 (0.66–0.89)		
	*T_1/2_ (h)*	2.21 (0.89–5.49)	1.15 (0.61–2.18)		
	*CL (L/h)*	-	-		
***p.o.***	*AUC_0–∞_* *(ng·h/mL)*	10.56 (6.77–16.48)	9.19 (5.76–14.69)	87 (48–157)	0.16
	*AUC_last_ (ng·h/mL)*	8.23 (6.15–11.02)	4.90 (3.53–6.80)	60 (40–89)	0.01
	*C_max_ (ng/mL)*	2.60 (2.13–3.18)	1.70 (1.44–2.01)	65 (52–83)	0.01
	*T_max_ (h)*	0.77 (0.65–0.91)	1.03 (0.84–1.27)		
	*T_1/2_ (h)*	2.54 (1.26–5.09)	4.83 (2.41–9.66)		
	*CL (L/h)*	-	-		

**MDZ**, midazolam; **APZ**, alprazolam; **CI**, confidence interval; AUCt0–tlast - area under the plasma concentration–time curve from time zero to the last sampled time point; AUCinf, AUC from time zero to infinity; **Cmax**, peak plasma concentration of the drug; **Tmax**, time needed to achieve Cmax.
