# Supplementary material for: Evaluation of 1β-Hydroxylation of Deoxycholic Acid as a Non-Invasive Urinary Biomarker of CYP3A Activity in the Assessment of Inhibition-Based Drug–Drug Interaction in Healthy Volunteers
Source: J Pers Med. 2021 May 24;11(6):457. doi: 10.3390/jpm11060457 (PMC8224742; doi:10.3390/jpm11060457)
Supplement: Supplementary file 1 [file jpm-11-00457-s001.zip › jpm-1201765-SI.pdf]

Supplementary Materials:

1 Pharmacokietics and Data Analysis of Midazolam Parameters

The pharmacokinetic (PK) parameters of MDZ were performed by standard noncompartmental methods using the WinNolin program, version 4.1 (Pharsight Corp, Mountain View, CA, USA). PK profiles were plotted as graphs of midazolam or 1-OH-midazolam concentration vs. time. The area under the curve (AUC) from time zero to the last sampling time point (AUCt) was computed using the log-linear trapezoidal rule. The linear trapezoidal rule was used for the ascending part of the PK curve, while the log trapezoidal rule was used for the descending part of the graph. The AUC extrapolated to infinite time was calculated as AUC_{inf} = AUCt + Ct/Ke where Ct is the concentration of the last sample taken and Ke is the terminal elimination rate constant. The terminal elimination rate constant Ke was determined by least squares regression analysis in the terminal phase of the semi log concentration-time curve. The half-life (t½) was calculated as t½ = 0.693/Ke.
Statistical analysis was performed using the GraFit software (version 3.0, Erithacus Software Limited, Middlesex, UK). The analysis included descriptive statistical, Kruskal-Wallis test, paired t-tests and the computation of the 90% confidence interval. The null hypothesis assumed no significant difference between the test and reference treatments. Analysis of variance (ANOVA) was performed on the AUC and Cmax after transformation of the data to their natural logarithmic (ln) values. The 90% confidence intervals (CIs) were calculated using the error variance obtained from ANOVA. The following equation was used:

$$90\%\ \mathrm{CI} = \left(\overline{\mathrm{X}}_{\mathrm{T}} - \overline{\mathrm{X}}_{\mathrm{R}}\right) \pm \mathrm{t}_{\mathrm{v}}^{0.1}\sqrt{S^2 x \frac{2}{n}}$$

where $\overline{X}_T$ and $\overline{X}_R$ are the geometric means of the ln transformed values for the test treatment (T) and the reference treatment (R); S^2 is the error variance obtained from the ANOVA; n is the number of participants, t is the t-value for 90% of the t-distribution and v is the degree of freedom of the error variance from the ANOVA. The anti-ln of the above CI values was then computed to give the 90% CIs of the ratios of the test to the reference treatment geometric means.

2. Pharmacokinetics of midazolam and metabolites

MDZ and 1′-OH-MDZ pharmacokinetics

Plasma concentrations of MDZ and its hydroxylated metabolite 1′-OH-MDZ were determined from 0 – 24 hrs post MDZ administration. The mean plasma concentration-time curve for MDZ alone and when co-administered with either fluconazole (FKZ), alprazolam (APZ) or itraconazole (ITZ) are presented in Fig S1.

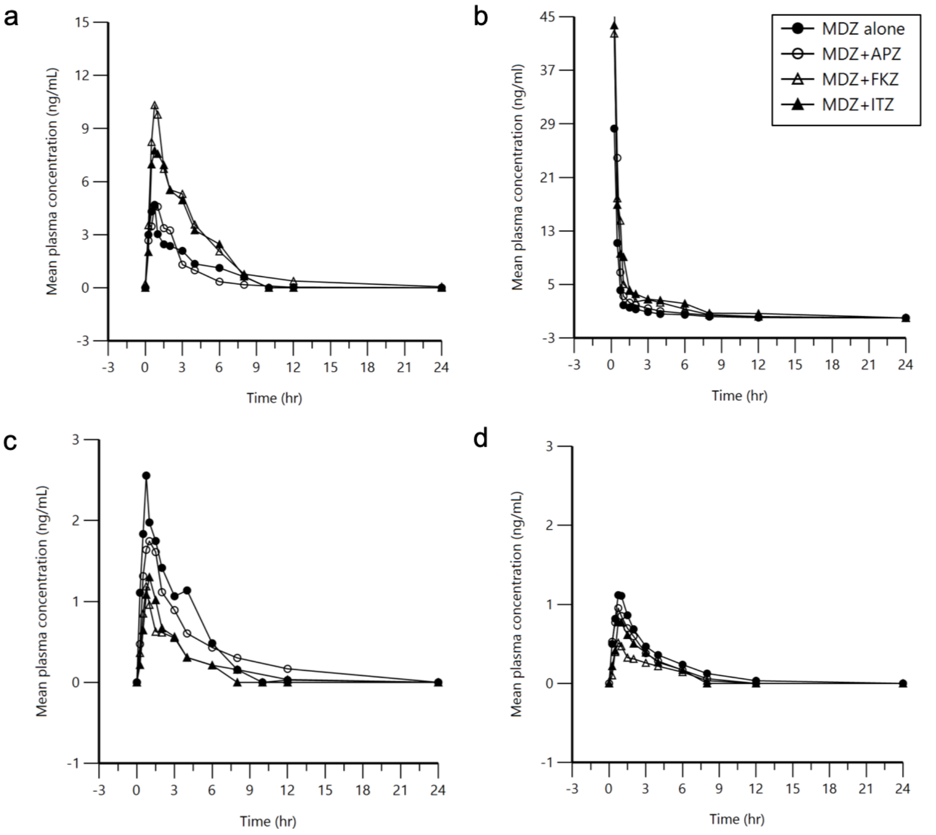

Figure S1: Mean plasma concentrations of midazolam (a and b) and 1′-hydroxymidazolam (c and d) versus time following administration of midazolam (1.5 mg p.o., a and c; or 1 mg i.v, b and d) alone and after chronic exposure with either APZ alprazolam (APZ), fluconazole (FKZ) or itraconazole (ITZ).

a. MDZ co-dosed with alprazolam

In the MDZ oral administration arm, neither MDZ nor 1′-OH-MDZ pharmacokinetic parameters were significantly altered by the 6-day APZ repeated dosing (Table S1). The percent geometric mean ratio for MDZ AUClast and Cmax were 104 (71 – 153) ($p = 0.23$) and 122% (89 – 166) ($p = 0.07$) respectively at 90 % confidence interval for orally administered MDZ. The AUClast geometric mean ratio was 148 (93 -234) ($p=0.12$) at 90% CI in the MDZ intravenous administration arm. The corresponding ratio of Cmax was however significant with a percent geometric mean of 193 (99 – 377) ($p = 0.02$) at 90% CI.

Table S1: Effect of alprazolam (1 mg) on midazolam and 1′-hydroxymidazolam pharmacokinetics following single dose administration (1.5 mg p.o or 1 mg i.v) in healthy participants (n = 10)

Parameter		Geometric mean values (90% CI)		Geometric Mean Ratio (90% CI)	p-value
		**MDZ alone**	**MDZ + APZ**		
**MDZ**					
i.v	*$AUC_{0-\infty}$ (ng·h/ml)*	16.78 (11.16 – 25.24)	26.25 (21.18 - 32.53)	156 (102 - 240)	0.09

	AUC_{last} (ng·h/ml)	16.64 (11.04 – 25.07)	24.57 (18.67 – 32.22)	148 (93 – 234)	0.12
	C_{max} (ng/ml)	25.98 (12.75 – 52.93)	50.71 (44.81 – 55.95)	193 (99 – 377)	0.02
	T_{max} (h)	0.29 (0.22 – 0.37)	0.30 (0.24 – 0.37)		
	$T_{1/2}$ (h)	2.06 (1.62 – 2.61)	3.73 (1.80 – 7.73)		
	CL (L/h)	59.88 (39.62 -89.50)	38.09 (30.73 -47.20)		
p.o.	$AUC_{0-\infty}$ (ng·h/ml)	10.92 (8.03 – 16.25)	10.99 (8.16 – 14.81)	100 (67 – 150)	0.31
	AUC_{last} (ng·h/ml)	10.43 (7.84 – 13.86)	10.85 (3.92 – 6.45)	104 (71 – 153)	0.23
	C_{max} (ng/ml)	4.13 (3.33 – 5.11)	5.03 (3.92 – 6.45)	122 (89 – 166)	0.07
	T_{max} (h)	0.76 (0.60 – 0.95)	1.03 (0.81 – 1.31)		
	$T_{1/2}$ (h)	1.63 (1.01 – 2.62)	1.47 (1.11 – 1.95)		
	CL (L/h)	137.34 (101 – 186)	136.46 (101 – 184)		
**1′-OH-MDZ**					
i.v	$AUC_{0-\infty}$ (ng·h/ml)	4.49 (2.12 – 9.50)	2.25 (1.07 – 4.72)	49 (18 – 100)	0.03
	AUC_{last} (ng·h/ml)	2.54 (1.49 – 4.35)	1.81 (0.84 – 3.86)	71 (28 – 179)	0.26
	C_{max} (ng/ml)	1.24 (1.02 – 1.50)	1.06 (0.88 – 1.27)	85 (67 – 109)	0.09
	T_{max} (h)	0.81 (0.65 – 1.10)	0.77 (0.66 – 0.89)		
	$T_{1/2}$ (h)	2.21 (0.89 – 5.49)	1.15 (0.61 - 2.18)		
	CL (L/h)	-	-		
p.o.	$AUC_{0-\infty}$ (ng·h/ml)	10.56 (6.77 – 16.48)	9.19 (5.76 – 14.69)	87 (48 – 157)	0.16
	AUC_{last} (ng·h/ml)	8.23 (6.15 – 11.02)	4.90 (3.53 - 6.80)	60 (40 – 89)	0.01
	C_{max} (ng/ml)	2.60 (2.13 – 3.18)	1.70 (1.44 – 2.01)	65 (52 – 83)	0.01
	T_{max} (h)	0.77 (0.65 – 0.91)	1.03 (0.84 – 1.27)		
	$T_{1/2}$ (h)	2.54 (1.26 – 5.09)	4.83 (2.41 – 9.66)		
	CL (L/h)	-	-		

MDZ, midazolam; **APZ**, alprazolam; **CI**, confidence interval; $AUCt_{0-tlast}$ - area under the plasma concentration–time curve from time zero to the last sampled time point; AUC_{inf}, AUC from time zero to infinity; **C_{max}**, peak plasma concentration of the drug; **T_{max}**, time needed to achieve C_{max}

b. MDZ co-dosed with fluconazole

Compared with MDZ administration alone, concomitant administration of MDZ (intravenous) with FKZ resulted in a 2-fold increase in the AUC_{last}, AUC_{∞} and the C_{max} (Table S2). This increase was significant as indicated by the 90% CI of the geometric ratio and well as the p-value ($p < 0.05$). The same pharmacokinetic parameters increased 1.5-fold for orally administered MDZ with no apparent effect on the $t_{1/2}$ and the t_{max}. The clearance was reduced by approximately 2-fold for both the intravenous and oral route. Parameters for the formation of 1′-OH-MDZ were reduced by approximately 50 and 40% for intravenous and orally administered midazolam respectively. They were however no changes in the t_{max} and $t_{1/2}$ for both administration routes.

Table S2: Effect of fluconazole (50mg) on midazolam and 1′-hydroxymidazolam pharmacokinetics following single dose midazolam administration (1.5 mg p.o or 1 mg i.v) in healthy participants (n = 10)

	Parameter	Geometric mean values (90% CI)		% Geometric Mean Ratio (90% CI)	p-value
		MDZ alone	MDZ + FKZ		
**MDZ**					
i.v	*$AUC_{0-\infty}$ (ng·h/ml)*	12.39 (8.18 – 18.76)	26.14 (16.48 – 41.46)	210 (117 – 377)	0.03
	AUC_{last} (ng·h/ml)	11.99 (8.02 – 18.76)	24.79 (15.74 – 39.05)	207 (117 – 365)	0.03
	C_{max} (ng/ml)	22.58 (19.68 – 25.9)	44.18 (38.69 – 50.44)	196 (164 – 234)	0.003
	T_{max} (h)	-	0.28 (0.23 - 0.35)	-	
	$T_{1/2}$ (h)	1.55 (1.03 – 2.34)	2.67 (1.87 – 3.81)	-	
	CL (L/h)	80.72 (53.31 – 122.22)	38.26 (24.12 -60.7)	-	
p.o.	*$AUC_{0-\infty}$ (ng·h/ml)*	19.38 (14.12 – 26.58)	30.81 (19.43 – 48.87)	159 (93 – 272)	0.08
	AUC_{last} (ng·h/ml)	18.51 (12.03 – 28.46)	26.79 (16.76 – 42.83)	145 (80 – 263)	0.08
	C_{max} (ng/ml)	6.18 (4.37 – 8.75)	9.27 (6.01 – 14.32)	150 (89 - 253)	0.05
	T_{max} (h)	0.79 (0.48 – 1.29)	0.67 (0.52 – 0.88)		
	$T_{1/2}$ (h)	2.26 (1.29 – 3.93)	2.66 (2.07 – 3.41)		
	CL (L/h)	77.41 (56.44 – 106.19)	48.68 (30.70 - 77.19)		
**1′-OH-MDZ**					
i.v	*$AUC_{0-\infty}$ (ng·h/ml)*	3.31 (1.92 – 5.72)	1.52 (0.82 – 2.83)	46 (21 – 100)	0.01
	AUC_{last} (ng·h/ml)	2.56 (1.65 - 3.96)	1.19 (0.68 – 2.10)	47 (24 – 91)	0.01
	C_{max} (ng/ml)	1.09 (0.76 – 1.56)	0.54 (0.38 – 0.77)	50 (31 – 77)	0.002
	T_{max} (h)	0.79 (0.69 – 0.90)	0.70 (0.59 – 0.83)	-	
	$T_{1/2}$ (h)	2.30 (1.44 -3.65)	2.25 (1.56 – 3.23)	-	
	CL (L/h)	-	-	-	
p.o.	*$AUC_{0-\infty}$ (ng·h/ml)*	6.73 (3.77 – 12.01)	2.80 (1.56 – 5.04)	42 (19 – 90)	0.05
	AUC_{last} (ng·h/ml)	6.56 (3.6 – 11.94)	2.43 (1.37 – 4.31)	37 (17 – 81)	0.04
	C_{max} (ng/ml)	2.81 (1.61 – 4.92)	1.05 (0.72 – 1.53)	37 (20 – 70)	0.03
	T_{max} (h)	0.83 (0.56 – 1.21)	0.83 (0.68 – 1)		
	$T_{1/2}$ (h)	1.99 (1.32 – 3.0)	1.71 (1 – 2.89)		
	CL (L/h)	**-**	**-**		

MDZ, midazolam; **FKZ**, fluconazole; **CI**, confidence interval; $AUCt_{0-tlast}$ - area under the plasma concentration–time curvefrom time zero to the last sampled time point; AUC_{inf}, AUC from time zero to infinity; **C_{max}**, peak plasma concentration of the drug; **T_{max}**, time needed to achieve C_{max}.

c. MDZ co-dosed with itraconazole

ITZ increased the AUC_{last}, AUC_{∞} and the C_{max} of intravenously administered MDZ by 2-fold (Table S3). This fold increase, reduction in t_{max}, prolongation of the half-life and reduction in clearance was comparable to what was observed for FKZ (Table S1 and S2). Effect of ITZ was however significant on orally administered MDZ with a percent 90% CI geometric mean ratio of 413 (239 – 713) (p=0.02) and 186 (127 –272) (p=0.02) for the AUC0–∞ and Cmax respectively. The clearance was reduced from 155.58 to 37.71 L/hr with the tmax being prolonged from 0.63 to 0.93 hrs. The differences in the formation of 1′-OH-MDZ was not significant between MDZ alone and compared to MDZ co-administered with ITZ for intravenously administered MDZ. There was however a significant difference in the $AUC_{0-\infty}$ for orally administered MDZ with percent geometric mean ratio of 53 (34 – 82) at 90 % CI (p = 0.01). The difference was however not significant for the Cmax with a geometric mean ratio of 65 (36 – 119) at 90 % CI (p = 0.15)

Table S3: Effect of itraconazole (100mg) on midazolam and 1′-hydroxymidazolam pharmacokinetics following single dose administration (1.5 mg p.o or 1 mg i.v) in healthy participants (n = 9)

	Parameter	Geometric mean values (90% CI)		Geometric Mean Ratio (90% CI)	p-value
		MDZ alone	MDZ + ITZ		
**MDZ**					
i.v	*$AUC_{0-\infty}$ (ng·h/ml)*	13.53 (11.09 – 16.5)	27.72 (15.34 -50.09)	205 (102 – 412)	0.09
	AUC_{last} (ng·h/ml)	12.88 (10.87 – 15.25)	24.01 (13.86-41.61)	187 (98 – 356)	0.10
	C_{max} (ng/ml)	25.01 (17.96 – 34.81)	43.00 (37.5 – 49.3)	172 (128 – 230)	0.01
	T_{max} (h)	0.28 (0.23 – 0.33)	0.27 (0.24 -0.30)		
	$T_{1/2}$ (h)	2.32 (1.62 – 3.31)	2.94 (2.1- 4.12)		
	CL (L/h)	73.92 (60.59 – 90.17)	36.07 (19.96 - 65.18)		
p.o.	*$AUC_{0-\infty}$ (ng·h/ml)*	9.64 (6.19 – 15.01)	39.78 (27.27 – 58.04)	413 (239 – 713)	0.02
	AUC_{last} (ng·h/ml)	8.61 (5.6 – 13.26)	25.13 (19.45 – 32.45)	292 (184 – 462)	0.01
	C_{max} (ng/ml)	4.86 (3.35 -7.04)	9.03 (7.46 – 10.92)	186 (127 – 272)	0.02
	T_{max} (h)	0.63 (0.42 – 0.93)	0.93 (0.66 – 1.3)		
	$T_{1/2}$ (h)	1.43 (1.10 -1.85)	3.46 (2.35 – 5.1)		
	CL (L/h)	155.58 (99.94 - 242.21)	37.71 (25.84 – 55.01)		
**1′-OH-MDZ**					
i.v	*$AUC_{0-\infty}$ (ng·h/ml)*	2.26 (1.5 – 3.41)	2.47 (1.34 – 4.58)	109 (53 – 224)	0.28
	AUC_{last} (ng·h/ml)	2.00 (1.41 – 3.01)	1.80 (1.20 – 2.67)	90 (54 – 149)	0.13
	C_{max} (ng/ml)	1.09 (0.87 – 1.35)	0.83 (0.67 -1.03)	76 (57 – 102)	0.02
	T_{max} (h)	0.79 (0.69 -0.90)	0.84 (0.77 – 0.92)		
	$T_{1/2}$ (h)	1.25 (0.8 – 1.95)	1.72 (0.95 – 3.1)		
	CL (L/h)	-	-		
p.o.	*$AUC_{0-\infty}$ (ng·h/ml)*	4.57 (3.04 – 6.88)	2.97 (1.82 – 4.85)	53 (34 – 82)	0.01
	AUC_{last} (ng·h/ml)	4.22 (2.8 -6.36)	2.44 (1.57 – 3.79)	58 (33 – 102)	0.04
	C_{max} (ng/ml)	2.29 (1.64 – 3.19)	1.21 (0.88 – 1.66)	65 (36 – 119)	0.15
	T_{max} (h)	0.71 (0.63 – 0.80)	0.90 (0.79 – 1.03)		
	$T_{1/2}$ (h)	1.34 (1.05 -1.7)	1.49 (0.95 – 2.34)		
	CL (L/h)	-	-		

MDZ, midazolam; **ITZ**, itraconazole; **CI**, confidence interval; $AUCt_{0-tlast}$ - area under the plasma concentration–time curve from time zero to the last sampled time point; AUC_{inf}, AUC from time zero to infinity; **C_{max}**, peak plasma concentration of the drug; **T_{max}**, time needed to achieve C_{max}
